# Supplementary material for: Real-life use of tocilizumab with or without corticosteroid in hospitalized patients with moderate-to-severe COVID-19 pneumonia: A retrospective cohort study
Source: PLoS One. 2021 Sep 10;16(9):e0257376. doi: 10.1371/journal.pone.0257376 (PMC8432821; doi:10.1371/journal.pone.0257376)
Supplement: S1 Table — (DOCX) [file pone.0257376.s001.docx]

**S1 Table**:

|  | **Multivariate analysis**^1^ | |
| --- | --- | --- |
|  | **HR** (95%CI) | *p-value* |
| **Composite outcome** | | |
| All participants (excluding 11 patients having received Remdesivir) | | |
| SoC | 1 (Ref) | - |
| CCS | 0.36 (0.09-1.49) | 0.157 |
| TCZ | 0.39 (0.34-0.45) | <0.001 |
| TCZ + CCS | 0.44 (0.37-0.52) | <0.001 |
| All participants according to number of TCZ infusion received (one or two) | | |
| SoC | 1 (Ref) | - |
| CCS | 0.40 (0.10-1.67) | 0.209 |
| TCZ (1 infusion) | 0.37 (0.31-0.44) | <0.001 |
| TCZ (2 infusions) | 0.58 (0.38-0.89) | 0.012 |
| TCZ (1 infusion) + CCS | 0.45 (0.22-0.91) | 0.027 |
| TCZ (2 infusions) + CCS | 0.58 (0.53-0.62) | <0.001 |

**Legend S1 Table:** ^1^ analysis adjusted for: age, gender, PO_2_/FiO_2_ ratio at baseline, CRP at baseline, Days from symptoms onset at the baseline, sum of comorbidities; SoC: Standard of Care; CCS: glucocorticosteroid; TCZ: tocilizumab.
