# Supplementary material for: Real-life use of tocilizumab with or without corticosteroid in hospitalized patients with moderate-to-severe COVID-19 pneumonia: A retrospective cohort study
Source: PLoS One. 2021 Sep 10;16(9):e0257376. doi: 10.1371/journal.pone.0257376 (PMC8432821; doi:10.1371/journal.pone.0257376)
Supplement: S2 Table — (DOCX) [file pone.0257376.s002.docx]

**S2 Table**

| **Country** | **Period** | **Outcome** | **Population study at baseline** | | | | | | **Concomitant CCS use** | **Main findings** | **Ref** |
| --- | --- | --- | --- | --- | --- | --- | --- | --- | --- | --- | --- |
|  |  |  | **n** | **Age, years^** | | | **% Males** | **COVID-19 severity** |  |  |  |
| US | March 1 to April 3, 2020 | 28-days mortality | 81  (TCZ^a^ only) | 64 [58-71] | | | 69.1% | Moderate (13.6%) to severe (86.4%) pneumonia | 70.4% | - Lower mortality if TCZ^a^ administered < 12 days after symptom onset (OR 0.296, 95%CI 0.098-0.889, p=0.04).  - No TCZ safety concern reported. | [33] |
| US | March 25 to April 24, 2020 | 7 and 14-days mortality | 66^b^  (TCZ^b^ only) | 61 [54.5-67] | | | 62.1% | Moderate (47%) to severe (25.7) to critical (27.3%) pneumonia | 66.7% | - No difference for mortality by day-7 (p=1) and day-14 (p=0.224) by comparing patients (critical vs moderate/severe at baseline).  - No TCZ ^b^ safety concern reported. | [42] |
| US | March 18 to May 2, 2020 | In-hospital mortality and IMV duration | 21  (TCZ^c^ only) | 56  [range 37-85] | | 67% | | Critical pneumonia (100% IMV) | 100%^X^ | - All patients received TCZ^c^ + CCS^X^: no deaths were reported, and 95% (20/21) of patients were extubated after a median of 8 (4-30) days.  - No TCZ safety concern reported. | [39] |
| Spain | March 12 to May 2, 2020 | In-hospital mortality (up to 80 days) | 77  (TCZ^d^ only) | 62  [range 53-72] | | 65% | | Moderate (67.5%) to severe (32.5%) pneumonia | N/A | - Lower mortality only if early response to TCZ^d^ assessed by BCRSS (OR 0.03, 95%CI 0.01-0.68, p=0.028).  - Among 10 deaths reported, 3 were COVID-related.  - No TCZ ^d^ safety concern reported. | [27] |
| Spain | March 16 to April 15, 2020 | Day-7 and day-14 clinical improvement | 88  (TCZ^e^ only) | 46.8±10.7 | | 65.9% | | Moderate (52.3%) to severe (44.3%) to critical (3.4%) pneumonia | 8% | - Clinical improvement by day 7 negatively correlated to baseline LDH > 450 U/L (OR 0.25, 95%CI 0.06‐0.99; *p=*0.048) or use of IFN‐β (OR 0.23, 95%CI 0.06‐0.94; *p*=0.041).  - No correlations at day-14.  - No TCZ^e^ safety concern reported. | [25] |
| Spain | March 27 to April, 2020 | In-hospital mortality, ICU admission | 72^c^  (TCZ^c^: 16, TCZ^c^ +CCS: 56) | 67.5 [61-76.7] | | 62.5% | | Moderate (22.2%) to severe (77.8%) pneumonia | 77.8%^XX^ | - Lower risk of death in TCZ^c^ + CCS^XX^ vs TCZ only (RR 0.20, 95%CI 0.08-0.47, p <0.01), but no influence on risk ICU admission (p=0.158)  - No TCZ safety concern reported. | [48] |
| Italy | N/A | 14-days clinical improvement (survival) | 63  (TCZ^f^ only) | 62.6±12.5 | | 88% | | Severe (95.2%) to critical (7.9%) pneumonia | N/A | - Higher likelihood of survival if TCZ^f^ administered < 6 days after hospital admission (HR 2.2, 95%CI 1.3-6.7, *p*<0.05).  - No TCZ ^f^ safety concern reported. | [34] |
| Italy | March 9 to 20, 2020 | 10-days clinical improvement | 100  (TCZ^g^ only) | 62 [57-71] | | 88% | | Severe (57%) to critical (43%) pneumonia | N/A | - Clinical and radiological improvement by BCRSS in 77% of patients, mortality 20%.  - Three cases of severe adverse events to TCZ^g^ reported | [35] |
| Italy | March 10 to 23, 2020 | 30-days mortality | 51  (TCZ^h^ only) | 60 I50-70] | | 78.4% | | Moderate (3.9%) or severe (84.3%) to critical (11.8%) pneumonia | N/A | - Higher 30-days mortality associated only to IMV at baseline (aHR 7.18, 95%CI 2-25, p=0.002) (Overall mortality 27%).  - Critically ill patients seem to have higher risk of bacterial superinfection related to TCZ ^h^ use. | [16] |
| US | March 1 to May 5, 2020 | 30-days mortality^@^ | 547^§^  SoC: 413  TCZ^i^:134 | | SoC: 69  [58-77]  TCZ^i^: 62  [53-70] | | SoC: 62%;  TCZ^i^:74% | SoC: Mild 42%,  Mod 31% Sev 26%  TCZ^i^: Mild 40.3%,  Mod 38.1%, Sev 21.6% | SoC: 63.7%  TCZ^1^: 59.7% | - Lower 30-days mortality, as statistical trend (p=0.053), in TCZ^i^ vs SoC (46% vs 56%) propensity score aHR 0.76, 95%CI 0.57-1.00).  - No TCZ^1^ safety concern. | [32] |
| US | March 1 to April 24,2020 | In-hospital mortality | 4,986  SoC: 3,076  TCZ^j^+ CCS^#^: 454  TCZ^j^: 73  CCS^#^:1,383 | | SoC: 64.6 [53-5-76.4]  TCZ^j^+ CCS^#^: 64.5 [54.9-73.1]  TCZ^j^: 62.4 [55 -68.7]  CCS^#^: 66.5 [55.8-76.9] | | SoC: 61%  TCZ^j^+ CCS^#^: 73%  TCZ^j^: 71%  CCS^#^: 65% | Data available for critical pneumonia (IMV) at baseline:  SoC: 5%  TCZ^j^+CCS^#^: 8%  TCZ^j^: 10%  CCS^#^: 6% | SoC: 0%  TCZ^j^: 0%  TCZ^j^+CCS^#^ & CCS^#^: 100% | - Lower risk for mortality in TCZ^j^+CCS^#^ vs SoC (HR 0.44, 95%CI 0.35-0.55, *p*<0.0001) and vs CCS^#^ group (HR 0.66, 95%CI 0.53-0.83, p=0.004).  - No significant difference for mortality in TCZ^j^ vs SoC (p=0.36)  - No TCZ^j^ safety concern. | [15] |
| US | March 9 to April 30,2020 | Mortality after OTI and at 28 days | 154  SoC: 76  TCZ^k^: 78 | | SoC: 60±14.5  TCZ^k^:55±14.9 | | SoC: 64%  TCZ^k^: 68% | Critical pneumonia (IMV) only* | SoC: 19.7%  TCZ^k^:29.5% | - Lower risk for mortality in TCZ^k^ vs SoC (aHR 0.55; 95%CI 0.33-0.90);  - Lower 28-days mortality in TCZ^k^ vs SoC (18% vs 36%, p=0.01);  - Higher risk for bacterial superinfection in TCZ^k^ vs SoC (45% vs 20%, p<0.001) | [37] |
| US | March 4 to May 10,2020 | In-hospital mortality | 3,924^§§^  SoC: 3,491  TCZ^j^: 433 | | SoC: 63  [52-72]  TCZ^j^: 58  [48-65] | | SoC: 62%  TCZ^j^: 69% | SoC: Mild 63%,  Mod 25% Sev 38%  TCZ^j^: Mild 35%,  Mod 18%, Sev 47% | SoC^£^:12.6%  TCZ^£,j^:18.7% | - Lower risk for mortality in TCZ^j^ vs SoC (aHR, 0.71; 95%CI 0.56-0.92) when TCZ^j^ given within 2 days after ICU admission.  - No TCZ^j^ safety concern. | [17] |
| Italy | February 26 to April 30,2020 | In-hospital mortality | 158  SoC: 68  TCZ^l^: 90 | | SoC: 71±14.6  TCZ^l^: 62.9±12.5 | | SoC: 72%  TCZ^l^:71% | SoC: Mild 29%,  Mod 55% Sev 16%  TCZ^l^: Mild 20%,  Mod 56%, Sev 24% | N/A | - Very lower mortality in TCZ^l^ vs SoC (7.7% vs 50%; aHR 0.057, 95%CI 0.017-0.187, *p*<0.001);  - No TCZ^l^ safety concern. | [26] |
| Italy | March 28 to April 21,2020 | 35-days survival | 80  SoC: 40  TCZ^m^: 40 | | SoC: 54.5 [50-73]  TCZ^m^:56 [50.3-73] | | SoC: 65%  TCZ^m^:65% | Severe pneumonia only* | SoC: 57.5%  TCZ^m^: 65% | - Lower mortality (5% *vs* 27.5%, *p*=0.006) and IMV (5% vs 30%, p=0.003) in TCZ^m^ vs SoC.  - No TCZ^m^ safety concern. | [30] |
| Italy | February 21 to April 30,2020 | Composite risk for IMV ± death at 14 days | 544  SoC: 365  TCZ^n^: 179 | | SoC: 69 [57-78]  TCZ^n^: 64 [54-72] | | SoC: 64%  TCZ^n^: 71% | Severe pneumonia only* | SoC: 17%  TCZ^n^: 30% | - Lower mortality in TCZ^5^ vs SoC: (7% vs 20%, p<0·0001**)**  - Lower risk for IMV ± death at 14 days in TCZ^n^ vs SoC (aHR 0.61, 95%CI 0.40-0.92; *p*=0·020)  - Higher risk for bacterial superinfection in TCZ^n^ vs SoC (13% vs 4%, p<0.0001) | [36] |
| Italy | May 13 to 19, 2020 | 28-days mortality | 65  SoC: 33  TCZ^o^: 32 | | SoC: 60 [50-75.5]  TCZ^o^: 64 [53-75] | | SoC: 82%  TCZ^o^: 91% | Severe pneumonia only* | SoC: 0%  TCZ^o^: 0% | - No reduction for 28-days mortality (p=0.15), nor for the risk for IMV (p=0.43) in TCZ^o^ vs SoC.  - No TCZ^o^ safety concern. | [43] |
| France | April 1 to May 11, 2020 | Composite risk for IMV ± death | 206  SoC: 176  TCZ^j^: 30 | | SoC: 74.3±11  TCZ^j^: 75.6±11 | | SoC: 59%  TCZ^j^: 70% | Moderate-to-severe pneumonia | SoC: 22%  TCZ^j^: 53% | - Lower risk for IMV or death in TCZ^j^ vs SoC (26.7% vs 52.3%, *p*=0.009), with difference for death not significant (p=0.253) whereas significant for IMV (0% vs 22.2%, p=0.004)  - No TCZ^j^ safety concern. | [47] |
| US | March 1 to April 22, 2020 | In-hospital mortality | 630^§§§,@@^  SoC: 420  TCZ^p^: 210 | | SoC: 65  [56-74]  TCZ: 62  [53-71] | | SoC: 67%  TCZ^p^: 74% | Critical pneumonia only* | SoC: 45%  TCZ^p^: 46% | - Lower mortality in TCZ^p^ vs SoC (49% vs 61%; HR 0.64, 95%CI 0.47-0.87, p=0.04), significant among patients <65 years old (HR0.64, 95%CI 0.44-0.93, p=0.023), and having CRP baseline>15 mg/dl (HR 0.48, 95%CI 0.30-0.77; p=0.0025). - Lower mortality in intubated patients in TCZ^p^ vs SoC (HR 0.63, 95%CI 0.46-0.85, p=0.003) - CCS not associated to lower mortality (HR 0.94, p=0.63) - No TCZ^p^ safety concern. | [38] |
| Sweden | March 11 to April 20, 2020 | 30-days mortality & clinical improvement | 87  SoC: 58  TCZ^q^: 29 | | SoC: 55  [52-65]  TCZ^q^: 58  [49-63] | | SoC: 78%  TCZ^q^: 97% | Critical pneumonia only* | SoC: 22%  TCZ^q^: 14% | - No differences for 30-days mortality (p=0.20) or IMV in TCZ^q^ vs SoC (p=0.41) - Lower ventilator-free days (p=0.04), length of ICU or hospital stay (p=0.04) in TCZ^q^ vs SoC - No TCZ^q^ safety concern. | [44] |
| US | March 19 to April 24, 2020 | 28 days clinical improvement and mortality | 88  SoC: 45  TCZ^r^: 43 | | N/A | | SoC: 69%  TCZ^r^: 70% | SoC: Sev 80%, Crit 20%  TCZ^r^: Sev 58%, Crit 42% | 0% | - No significant differences for 28-days mortality (0.26) or clinical improvement (p=0.85) in TCZ^r^ vs SoC - No significant TCZ^r^ safety concern. | [45] |
| Spain | January 31 to April 23, 2020 | In-hospital mortality | 1,229  SoC: 969  TCZ^s^: 260 | | SoC: 68  [57-80]  TCZ^s^: 65  [565-76] | | SoC: 59%  TCZ^s^: 73% | N/A | SoC: 35%  TCZ^s^: 93% | - Lower mortality only with CRP baseline>15 mg/dl in TCZ^s^ vs SoC (aHR 0.34, 95%CI: 0.17-0.71, p=0.005) - No significant TCZ^r^ safety concern. | [41] |
| Italy | March 11 to April, 2020 | 30-day survival (no IMV or death) | 196  SoC: 66  CCS^$^: 45  TCZ^t^: 29  TCZ^t^ +CCS^$^:56 | | SoC: 73.5±14  CCS^$^: 67.5±14  TCZ^t^: 66±10  TCZ^t^ +CCS^$^: 61±11.7 | | SoC: 62%  TCZ^t^/CCS^$^/TCZ^t^+ CCS^$^: 70% | Mod: 16.3%  Sev: 83.7% | SoC: 0%  TCZ^t^: 0%  TCZ^t^+CCS^$^ &CCS^$^: 100% | - Lower risk for 30 days IMV or death (aHR 0.48, 95%CI: 0.23-0.99, p=0.049) and higher overall survival (aHR 0.41, 95%CI: 0.19-0.89, p=0.025) in TCZ^t^/CCS^$^/ TCZ^t^+CCS^$^ vs SoC (no difference comparing TCZ vs CCS vs TCZ+CCS) - Slightly higher incidence of secondary bacterial infections and reversible transaminitis in non-SoC vs Soc, although no comparisons performed | [29] |
| Italy | March 13 to April 3, 2020 | Overall survival | 222  SoC: 148  TCZ: 74 | | SoC: 59  [52-70]  TCZ^u^: 59  [51-71] | | SoC: 81%  TCZ^u^: 82% | SoC: Sev 30.4%,  Crit 69.6%  TCZ^u^: Sev 20.3%, Crit 79.7% | 0% | - Higher overall survival in TCZ^u^ vs SoC (HR 0.49, 95%CI: 0.26-0.95, p=0.035), but in critical disease only - TCZ safety concerns reported | [40] |
| US | March 1 to May 5, 2020 | In-hospital mortality | 132  SoC: 66  TCZ^v^: 66 | | SoC: 61±16  TCZ^v^: 62±13 | | SoC: 76%  TCZ^v^: 70% | Severe pneumonia only* | SoC: 7%  TCZ^v^: 18% | - No difference for mortality in TCZ^v^ vs SoC (p=1.00) No TCZ^v^ safety concern. | [28] |
| France | March-April 2020 | 28-days IMV or death | 168  SoC: 84  TCZ^r^: 84 | | SoC: 64±17  TCZ^r^: 65±13 | | SoC: 58%  TCZ^r^: 65% | Severe pneumonia only* | SoC: 29%  TCZ^r^: 31% | - Lower risk for 28 days IMV or death (aHR 0.49, 95%CI: 0.3-0.81, p=0.005) and of 28-days death (aHR 0.42, 95%CI: 0.22-0.82, p=0.008) in TCZ^r^ vs SoC - No TCZ^r^ safety concern. | [31] |
| US | March 10 to April 2, 2020 | 30-days death after intubation | 115  SoC: 70  TCZ^z^: 45 | | SoC: 60.6±13  TCZ^z^: 56±14.7 | | SoC:73%  TCZ^z^: | Critical pneumonia onlt* | SoC: 78.6%  TCZ^z^: 73.3 | - No difference for 30-days mortality among patients receiving IMV (OR 1.04, 95%CI: 0.27-3.75) - No TCZ^z^ safety concern. | [46] |

**Legend S2 Table:** ^ data as median [IQR] or mean (±SD). CCS: corticosteroid; TCZ: Tocilizumab; IFN: Interferon; SoC: Standard of Care; IMV: Invasive Mechanical Ventilation; ICU: Intensive Care Unit; N/A: Not available; Mod: moderate COVID; Sev: Severe COVID; Crit: Critical COVID; OR: Odds Ratio; HR: Hazard ratio; aHR: adjusted Hazard Ratio; RR: Relative Risk; IQR: interquartile range; BCRSS: Brescia COVID Respiratory Severity Scale (*Toniati P, 2020*).

^X^ Methylprednisolone administered after TCZ, 11h (1.5-141h) after intubation, 125 mg x 4 the 1^st^ day, then 60 mg x 2/day and tapering over 10 days; ^XX^ Methylprednisolone: 250 mg loading-dose, then 40 mg x 2/day for 5 days, then stop without tapering; ^#^ Dexamethasone, methylprednisolone; ^£^ Methylprednisone or prednisone; ^$^ Methylprednisone 1 mg/kg/day for 5 days, then 0.5 mg/kg/day for 5 days; * data on both study group (TCZ & SoC); ^@^ exploratory objective (secondary objective) of the study; ^@@^ propensity-score matched population; ^§^ all treated in ICU setting (25% of participants being severely ill at baseline); ^§§^ all treated in ICU setting (38.9% of participants being severely ill at baseline); ^§§§^ all ICU-patients (93% of them intubated at some points)

^a^ TCZ iv 8 mg/kg, once (76.5%) or twice (23.5%); ^b^ TCZ iv 8 mg/kg once; ^c^ TCZ iv 400 mg once; ^d^ TCZ iv 600 mg 1^st^ dose, with 400 mg 2^nd^ or 3^rd^ dose; ^e^ TCZ iv 600 mg 1^st^ dose, 400 mg other doses, once (8%), twice (63.8%), thrice (28.4%); ^f^ TCZ iv 8 mg/kg or sc 324 mg, once (17.5%) or twice (82.5%); ^g^ TCZ iv 8 mg/kg, twice (87%) or thrice (13%); ^h^ TCZ iv 400 mg twice 12h apart (35%) or 8 mg/kg twice 12h apart (65%); ^i^ TCZ iv 400 mg once (78%); ^j^ unspecified TCZ dose, number of doses, way of administration; ^k^ TCZ iv 8 mg/kg once (94.9%) or twice (5.1%); ^l^ TCZ once, 400 mg iv (47.7%) or 324 mg sc (52.3%); ^m^ TCZ sc 324 mg twice; ^n^ TCZ twice 12h apart: iv (8 mg/kg) (49.2%) or sc (324 mg) (50.8%); ^o^ TCZ iv 400 mg once (72%) or twice (28%); ^p^ TCZ iv 400 mg once (88%) or twice (12%); ^q^ TCZ iv 8 mg/kg once; ^r^ TCZ 400 mg iv once; ^s^ TCZ iv 600-800 mg once; ^t^ TCZ iv (57.6%) or sc (42.4%), once (87%) or twice (13%); ^u^ TCZ iv 8 mg/kg once or twice (proportions unspecified); ^v^ TCZ iv 400 mg (80%) or 600-800 (20%) once; ^z^ TCZ iv 4.8 mg/kg once.
