# Supplementary material for: Real-life use of tocilizumab with or without corticosteroid in hospitalized patients with moderate-to-severe COVID-19 pneumonia: A retrospective cohort study
Source: PLoS One. 2021 Sep 10;16(9):e0257376. doi: 10.1371/journal.pone.0257376 (PMC8432821; doi:10.1371/journal.pone.0257376)
Supplement: S3 Table — (DOCX) [file pone.0257376.s003.docx]

**S3 Table**

| **Country** | **Period** | **RCT** | **Outcome** | **Population study at baseline** | | | | **Concomitant CCS use** | **Main findings** | **Ref** |
| --- | --- | --- | --- | --- | --- | --- | --- | --- | --- | --- |
|  |  |  |  | **n** | **Age, years^** | **Males, %** | **COVID-19 severity** |  |  |  |
| Italy^1^ | March 11 to June 11, 2020 | Open-label | IMV or death or clinical worsening at 14 days (and 30-days for IMV or death) | 123  TCZ: 60  SoC: 63 | 60 [53-72] | 61.1% | Moderate pneumonia with PaO_2_/FiO_2_ between 200 and 300 | 0%^§^ | *Trial early stopped for futility*  - No significant differences (p=0.87) for clinical worsening (28.3% vs 27%), IMV (10% vs 7.9%) or death (1.7% vs 1.6%) at 14-days (and at 30-days for death and/or mortality) between groups.  - No TCZ safety concern reported. | [18]^1^ |
| US^2^ | April 20 to June 15, 2020 | Double blinded, placebo-controlled | IMV or death at 28 days, clinical worsening at 14 days | 243  TCZ: 161  Placebo: 81 | 59.8  [45.3-69.4] | 58% | Moderate pneumonia | TCZ: 11%  Placebo: 6% | - No significant difference between groups for IMV or death at 28-days (aHR 0.66, 95%CI 0.28-1.52) or clinical worsening (aHR 0.88, 95%CI 0.45-1.72) at 14 days  - Severe neutropenia more frequent in TCZ group (p=0.002), whereas infections were more frequent in placebo arm (p=0.03) | [19]^2^ |
| Multiple countries^3*^ | N/A | Double blinded, placebo-controlled | IMV or death at 28 days | 377  TCZ: 249  Placebo:128 | 55±14.4 | 59.2% | Moderate pneumonia | TCZ: 80%  Placebo: 87% | - Lower IMV or death at 28 days in TCZ vs placebo group (12% vs 19%; HR 0.56, 95%CI 0.33-0.97)  - No TCZ safety concern reported | [20]^3^ |
| France^4^ | March 31 to April 18, 2020 | Open-label° | NIV or IMV or death at 14 days | 131  TCZ: 64  SoC: 67 | 64  [57.1-74.3] | 68% | Moderate-to-severe pneumonia | TCZ: 33%  SoC: 61% | Lower NIV or IMV or death at 14-days in TCZ vs SoC group (median posterior HR 0.58, 90%CrI 0.33-1.00)°, but no difference between groups for death at 28-days (aHR, 0.92; 95%CI 0.33-2.53)°  - No TCZ safety concern reported | [21]^4^ |
| Brazil | May 8 to July 17, 2020 | Open-label, superiority trial | Clinical status (including NIV or IMV or death) at 15-days and 28-days | 129  TCZ: 65  SoC: 64 | 57±14 | 68% | Severe-to-critical pneumonia | TCZ: 84%  SoC: 89% | *Trial early stopped for futility*.  - No significant differences for IMV or death at 15-days between groups (p=0.32)  - Higher 28-days mortality in TCZ group (OR: 2.7, 95%CI 0.97-8.35, p=0.07) as statistical trend  - No TCZ safety concern reported | [22] |
| Multiple countries^5#^ | April 19 to Nov 19, 2020 | Open-label° | respiratory & cardiovascular organ support-free days up to day 21; in-hospital survival | 755  TCZ: 353  SoC: 402 | TCZ: 61.5±12.5  SoC: 61.1±12.8 | TCZ: 74%  SoC: 70% | TCZ: Severe (70.2%) or Critical (29.5%) pneumonia  SoC: Sever (69.4%) or Critical (30.1%) pneumonia | TCZ: 85.5%  SoC: 85.8% | - Higher organ support-free days in TCZ vs SoC (aOR 1.64, 95%CrI 1.25, 2.14, posterior probability of superiority >99.9%); - Higher in-hospital survival in TCZ vs SoC (aOR 1.64 95%CrI 1.14, 2.35) posterior probability of superiority 99.6%); - No TCZ safety concern reported. | [23]^5^ |
| Multiple countries^6†^ | April 3 to May 28, 2020 | Double blinded, placebo-controlled | Clinical status (including death) at 28 days | 438  TCZ: 294  Placebo:144 | TCZ: 60.9±14.6  Placebo: 60.6±13.7 | 70% | Severe pneumonia | TCZ: 19.4%  Placebo: 28.5% | - No difference for clinical improvement at 28 days (p=0.3) - No difference for mortality at 28 days in TCZ vs placebo (34.9% vs 38.5%, p=0.94) - No TCZ safety concern reported | [24]^6^ |

**Legend S3 Table:** TCZ Tocilizumab; SoC: Standard of Care; CCS: Glucocorticosteroid; Sev.: Severe COVID-19; Crit. Critical COVID-19; HR: Hazard ratio; aHR: adjusted Hazard ratio OR: Odds ratio; CI: Confidence interval; CrI: credible interval (Bayesan statistic); IMV: Invasive Mechanical Ventilation; NIV: non-invasive ventilation;

^ data as median [IQR] or mean (±SD); ^§^ No patients were taking glucocorticosteroid before enrolment, but no data were shown for their use in both groups after randomization; * US, Mexico, South Africa, Kenya, Peru, Brazil; ° Bayesan statistical approach; ^#^ 113 clinical sites from different countries (Australia, Belgium, Canada, Croatia, Finland, France, Germany, Hungary, India, Ireland, Nepal, Netherland, New Zeland, Pakistan, Portugal, Romania, Saudi Arabia, Spain, UK, US); ^†^ 62 clinical sites in 9 countries (Canada, Denmark, France, Germany, Italy, the Netherlands, Spain, UK, US).

^1^ RCT-TCZ-COVID-19; ^2^Boston Area COVID-19 Consortium (BACC) Bay Tocilizumab; ^3^ EMPACTA; ^4^ CORIMUNO-19; ^5^ REMAP-CAP;

^6^ COVACTA
