# Supplementary material for: Real-life use of tocilizumab with or without corticosteroid in hospitalized patients with moderate-to-severe COVID-19 pneumonia: A retrospective cohort study
Source: PLoS One. 2021 Sep 10;16(9):e0257376. doi: 10.1371/journal.pone.0257376 (PMC8432821; doi:10.1371/journal.pone.0257376)
Supplement: Attachment — Submitted filename: Response to reviewers.docx [file pone.0257376.s005.docx]

**Title:**

**Real-life use of tocilizumab with or without corticosteroid in hospitalized patients with moderate-to-severe COVID-19 pneumonia: a retrospective cohort study (PONE-D-21-08792)**

Response to the reviewers’ comment:

First of all, we would like to thank the reviewers for their comment. Below, our reply to the points raised by the reviewers:

3. Have the authors made all data underlying the findings in their manuscript fully available?

Reviewer #1: Yes

Reviewer #2: No

Authors: We added a file with all data underlying our findings as supplementary material and we added the sentence “All relevant data are within the manuscript and its Supporting Information files”.

Reviewer#2:

1. The overall idea of demonstrating the benefit of TCZ in addition to other standard therapy, in a group of patients who are extremely diverse in terms of severity and nature of the illness is extremely difficult.

Although the authors did acknowledge this with a statement in the Discussion Pg 2- "It is important to highlight that clinical management of COVID-19 hospitalized patients is complex and not standardized also for the respiratory support" - the authors could elaborate more on how the clinical management was standardised with perhaps a hospital policy with the assistance of a standard algorith or flow chart that could pose some level of

standardisation in the treatment.

Authors reply: the sentence considered was not referred to our setting, but it was in general. Although that, we agree with the reviewer and we have modified the mentioned sentence and added a new one as follows: “*In general, it is important to highlight that the clinical management of COVID-19 hospitalized patients is complex and not standardized also for the respiratory support, with SoC being possibly different among clinical centres and overtime. In order to better standardize the clinical management of our patients, a simplified flow-chart for internal use was established*”.

2. In the limitation section the authors should include the small numbers that could limit the validity of the results.

A: the small number of patients has been added as study limitation as requested
