# Supplementary material for: Real-life use of tocilizumab with or without corticosteroid in hospitalized patients with moderate-to-severe COVID-19 pneumonia: A retrospective cohort study
Source: PLoS One. 2021 Sep 10;16(9):e0257376. doi: 10.1371/journal.pone.0257376 (PMC8432821; doi:10.1371/journal.pone.0257376)
Supplement: Attachment — Submitted filename: Response to reviewer_PONE-D-21-08792R1.docx [file pone.0257376.s006.docx]

**RESPONSE TO REVIEWER**

(19th August 2021)

**Reviewer #3:**

the paper is interesting in its concept and being based on "real life experience', the data offers a

different prospective about use of TCZ either alone or in conjunction with GCs, as the published results about this drug is controversial. however, this manuscript could benefit from some modifications to pinpoint its significant results.

**Abstract:**

**Q1.** In conclusion subsection, re-write it to be more concise and to the point, according to your results does use of TCZ alone was beneficial to patients or adding GCS was more beneficial?

**A1.** Modified as requested

**Introduction**:

**Q2.** It is apparent that this study took place in Italy, please add sentence referring to death rate by age in Italy (if available)

**A2.** We thank the reviewer for the suggestion. Data (with the corresponding reference) have been changed

**Q3.** in the first paragraph discussing cytokines, reference no. 8 reported low level of IL-6 in association with critical COVID-19, please add more rationale for optioning to use drug act against IL-6 receptors (i;e bolster what is written in references 6,9 and 10).

**A3.** Reference n.8 is a review comparing IL-6 serum level in severe COVID-19, hyperinflammatory ARDS, sepsis, and CRS in the setting of CAR-T cells Therapy. The paper shows that the peak of serum IL-6 in severe COVID-19 was lower than what observed in the other conditions known to be associated to high serum level of IL-6. Tocilizumab is already authorized for the treatment of the CRS in the setting of CAR-T cells Therapy. We thanks the reviewer for its suggestion and, in order to avoid misinterpretation, the sentence has been modified focusing on the lower peak of IL-6 in severe COVID-19 in comparison to the other clinical conditions that have been specified in the manuscript.

**Q4.** does the presence of more virus variants affects the efforts to establish standardized therapy? please clarify in introduction.

**A4.** We thank the reviewer for the suggestion, but our paper did not take in account viral variants (and their potential impact on pathogenesis and treatment) because that is beyond the objective of our study.

**Q5.** sentence linked to reference 11 is ambiguous, please clarify.

**A5.** The sentence related to ref 11 is about the results of the RECOVERY trial in which the reduced mortality related to the corticosteroid therapy was evident only in patients in need of oxygen support (being possibly harmful in those receiving CCS and not in need of oxygen supply (RR 1.19))

**Q6.** please add three-sentences paragraph about TCZ, the cornerstone of this study.

**A6.** The sentences have been added as requested

**Method section:**

**Q7.** Figure 1 needs more bolstering, it would be best if the flowchart contains original enrolled number, number of patients that were excluded and their exclusion criteria, eligible enrolled patients and their inclusion criteria, distribution of patients on groups with subdivision number of deaths in every group, number of patients discharged alive, number of patients who needed ICU.

**A7.** We have modified the figure 1 taking in account the reviewer suggestions and we have also better specified inclusion criteria in the main manuscript

**Q8.** In SOC, does this hospital/region/national guidelines, please clarify as a footnote.

**A8.** SoC was based on advices (not properly guidelines) from a panel of experts of the Italian Society of Infectious and Tropical Diseases (SIMIT, Società Italiana di Malattie Infettive e Tropicali) and this has been added in the text

**Q9.** the dose of TCZ is somewhat different from the one used in Rashad et al., 2021 and Albertini et al., 2021, does the different dosage affected the measured outcomes/survivability?

**A9.** We agree with the reviewers about the heterogeneity of the dosage (and ways of administration: sc, iv) of TCZ used in different studies and we cannot answer properly to this question because, as in our study, no Therapeutic Drug Monitoring has been done as in all other published studies on TCZ use in COVID-19. Pharmacokinetic studies on TCZ are available only among few rheumatologic patients (and healthy controls), but not in COVID-19 patients. Furthermore, in order to be more precise on this aspect (and showing the heterogeneity among studies), in Table S2 which resumes observational studies on TCZ in COVID-19, the doses and ways of administration have been already specified as footnote of the table for each study: this aspect has been also already raised in the discussion section.

**Q10.** authors mentioned "the majority of patients received methylprednisolone, please add the exact number, and what about he remaining patients in the group, did they receive dexamethasone? please clarfiy.

**A10.** As suggested by the reviewer, the proportion of patients taking methylprednisone or other CCS (dexamethasone) has been specified in the manuscript.

**Results**:

**Q11.** group classification belongs to method section, please amend.

**A11.** Modified as suggested.

**Q12.** does the co-morbidity at admission affected the survival rate?

**A12.** Yes, the coefficient of the sum of comorbidities was significant in the fully adjusted survival analysis at 14-days (HR 1.23 95%CI: 1.10-1.38, p<0.001) and 28-days (HR 1.20, 95%CI: 1.10-1.32, p<0.001). A specific sentence has been added in the results section.

**Q13.** results in its current format are confusing, please reorder to pinpoint the most significant results of the study. Start with epidemiological data (age, co-morbidities, time between appearance of signs and admission, signs at admission) followed by baseline parameters, treatment groups and parameters, adverse effects and survival rate and end with multivariate COX hazard regression model.

**A13.** We thank the reviewer for the suggestion that we have followed by modifying the organization of the results as requested

**Q14.** no need to repeat drugs used in SOC protocol in results section.

**A14.** Modified as suggested

**Q15.** this paragraph " intubation occurs more frequently in TCZ group, 2/3 with TCZ+ GCs), please clarify this result, which group exactly? and what number "26" stands for?

**A15.** We observed 26 cases oro-tracheal intubation: 17 in TCZ+CCS and 7 in TCZ group. The sentence has been modified as requested.

**Discussion**:

**Q16.** although there were conflicting results about usage of TCZ, it was not discussed extensively, please amend.

**A16.** We did not agree with this observation. Results of many different studies on TCZ use in COVID-19 (all resumed in Table S2 and S3) have been discussed, as well as factors hampering results comparison between and among studies.

**Q17.** also, based on your results, which was more beneficial, TCZ alone, TCZ with GCs or SOC alone? please discuss does different GCs used affected the results outcomes?

**A17.** As already stated in the discussion section of the manuscript, the co-administration of corticosteroid was protective without improving tocilizumab outcomes effect, excepting in cases with more severe respiratory disease. This concept has been added in the discussion as requested. Moreover, as requested, it has been specified that the use of different corticosteroid molecules (methylprednisolone and dexamethasone) did not changed study outcomes

**Q18.** does co-morbidities affect outcome regardless of used therapeutic regimens which co-morbidity benefit/ worsen by TCZ either alone or in conjunction with GCS?

**A18.** Although interesting, we did not test an hypothesis for the interaction between co-morbidities and treatment groups because it was beyond the study objectives. Moreover, we do not think that the number of patients in some of the groups is enough to perform this analysis

**Conclusion**

**Q19.** it is preferable if conclusion does not contain references, please re-write this section and use "take home message" of your most significant results and recommendations based on your results

**A19.** We thank the reviewer for the suggestion, and we have modified the text accordingly
